# Detection of SARS-CoV-2 RNA by multiplex RT-qPCR

**DOI:** 10.1371/journal.pbio.3000867

**Published:** 2020-10-07

**Authors:** Eriko Kudo, Benjamin Israelow, Chantal B. F. Vogels, Peiwen Lu, Anne L. Wyllie, Maria Tokuyama, Arvind Venkataraman, Doug E. Brackney, Isabel M. Ott, Mary E. Petrone, Rebecca Earnest, Sarah Lapidus, M. Catherine Muenker, Adam J. Moore, Arnau Casanovas-Massana, Yale IMPACT Research Team, Saad B. Omer, Charles S. Dela Cruz, Shelli F. Farhadian, Albert I. Ko, Nathan D. Grubaugh, Akiko Iwasaki

**Affiliations:** 1 Department of Immunobiology, Yale School of Medicine, New Haven, Connecticut, United States of America; 2 Department of Internal Medicine, Section of Infectious Diseases, Yale School of Medicine, New Haven, Connecticut, United States of America; 3 Department of Epidemiology of Microbial Diseases, Yale School of Public Health, New Haven, Connecticut, United States of America; 4 The Connecticut Agricultural Experiment Station, Department of Environmental Sciences, New Haven, Connecticut, United States of America; 5 Yale Institute of Global Health, New Haven, Connecticut, United States of America; 6 Yale School of Nursing, New Haven, Connecticut, United States of America; 7 Section of Pulmonary, Critical Care, and Sleep Medicine, Department of Internal Medicine, Yale School of Medicine, New Haven, Connecticut, United States of America; 8 Howard Hughes Medical Institute, Chevy Chase, Maryland, United States of America; University of Wisconsin-Madison, UNITED STATES

## Abstract

The current quantitative reverse transcription PCR (RT-qPCR) assay recommended for severe acute respiratory syndrome coronavirus 2 (SARS-CoV-2) testing in the United States requires analysis of 3 genomic targets per sample: 2 viral and 1 host. To simplify testing and reduce the volume of required reagents, we devised a multiplex RT-qPCR assay to detect SARS-CoV-2 in a single reaction. We used existing N1, N2, and RP primer and probe sets by the Centers for Disease Control and Prevention, but substituted fluorophores to allow multiplexing of the assay. The cycle threshold (Ct) values of our multiplex RT-qPCR were comparable to those obtained by the single assay adapted for research purposes. Low copy numbers (≥500 copies/reaction) of SARS-CoV-2 RNA were consistently detected by the multiplex RT-qPCR. Our novel multiplex RT-qPCR improves upon current single diagnostics by saving reagents, costs, time, and labor.

## Abstract 2

The current RT-qPCR assay recommended for SARS-CoV-2 testing in the United States requires analysis of three genomic targets per sample: two viral and one host. This study describes a multiplex PCR that saves both reagents and labor while accurately detecting SARS-CoV-2 RNA.

## Introduction

The ongoing global pandemic caused by severe acute respiratory syndrome coronavirus 2 (SARS-CoV-2) and associated coronavirus disease 2019 (COVID-19) has caused more than 25 million infections and killed more than 850,000 people as of September 2, 2020, and the virus continues to spread throughout the globe [1]. In the absence of a specific vaccine or effective therapy for the treatment of COVID-19, public health infection prevention measures, including contact tracing and isolation measures, are currently our only tool to stem transmission. However, testing, contact tracing, and isolation measures require rapid and widespread testing. Here, we improved a quantitative reverse transcription PCR (RT-qPCR) assay for the detection of SARS-CoV-2 to allow for more rapid and widespread testing.

While a number of primer and probe sets for the detection of SARS-CoV-2 RNA by RT-qPCR have become available since the identification of this novel virus, their broad deployment has been hampered partially by the availability of testing reagents. The current RT-qPCR assay developed by the Centers for Disease Control and Prevention (CDC) targets 2 different conserved segments of the viral nucleocapsid gene (N1 and N2) as well as the human RNase P gene as a sampling control [2]. This protocol therefore requires 3 reactions to be performed per patient sample, which, in addition to requiring a large amount of resources, also increases the chance for error. In an effort to reduce reagents, time, potential error, and labor per sample, we devised a multiplex RT-qPCR for the detection of SARS-CoV-2. To do this, we utilized the existing N1 and N2 primer and probe sets published by the CDC; however, we substituted different fluorophores to enable multiplexing. We found the accuracy and specificity of this method to be similar to those of single RT-qPCR. Therefore, this novel multiplex RT-qPCR assay provides equivalent diagnostic accuracy to current single methods in fewer reactions and utilizes less reagents and time.

## Results

### Determination of lower limit of virus concentration detected by multiplex RT-qPCR

**Fig 1:**
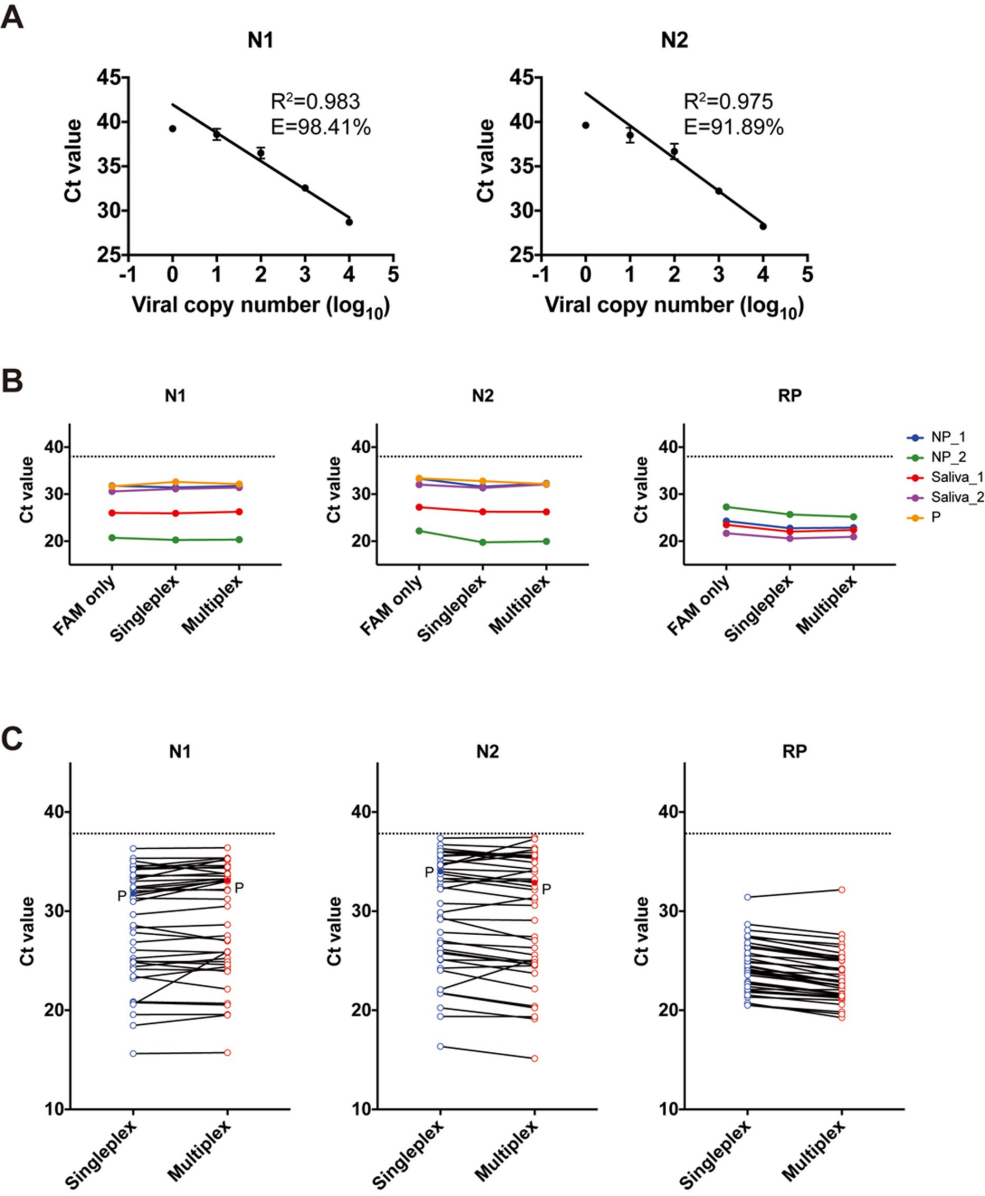
The qualification of multiplex RT-qPCR for SARS-CoV-2 compared with single RT-qPCR. (A) Multiplex RT-qPCR detection of SARS-CoV-2 N1 and N2 genes was validated using 10-fold dilutions of viral RNA into pooled negative NP samples. We measured sensitivity and efficiency for 20 replicates. Data are mean ± SD. Individual values are indicated in Table 1. (B) The Ct values for 4 independent COVID-19 inpatients’ NP (*n* = 2) or saliva (*n* = 2) samples, 1 negative control, and 1 positive control (P) (10^3^ virus copies/μl) were compared between single RT-qPCR (FAM only), multicolor single RT-qPCR (Singleplex), and multiplex RT-qPCR (Multiplex). The dotted line indicates the cutoff Ct value of 38. Negative control was undetectable. Individual values are indicated in Table 2. (C) Forty-two RNA templates from NP swabs and saliva samples obtained from COVID-19 inpatients or healthcare workers and positive control (P) (10^3^ virus copies/μl) were investigated via single and multiplex RT-qPCR. The dotted line indicates the cutoff Ct value of 38. Individual values are indicated in Table 3. COVID-19, coronavirus disease 2019; Ct, cycle threshold; E, amplification efficiency; NP, nasopharyngeal; P, positive control; *R*^2^, regression coefficient value; RT-qPCR, quantitative reverse transcription PCR; SARS-CoV-2, severe acute respiratory syndrome coronavirus 2.

The limit of detection (LOD) was analyzed using 10-fold serial dilutions of full-length SARS-CoV-2 RNA into RNA extracted from pooled nasopharyngeal swabs from SARS-CoV-2-negative human samples. The cycle threshold (Ct) values and detection rates are shown in Table 1. The slope of the standard curves for N1 and N2 were −3.36 and −3.52, respectively. The amplification efficiency was above 90% for both primer–probe sets (Fig 1A). All primer–probe sets and conditions were able to detect SARS-CoV-2 at 500 virus copies per reaction (Table 1). These data are consistent with previous studies [3,4].

**Table 1:** Lower limit of detection of SARS-CoV-2 in multiplex RT-qPCR.

Copies/reaction	Ct: average (SD)		Detected/tested (%)	
	N1	N2	N1	N2
5	39.24 (0.38)	39.63	3/20 (15)	1/20 (5)
50	38.59 (0.64)	38.51 (0.84)	12/20 (60)	5/20 (25)
500	36.50 (0.63)	36.67 (0.87)	20/20 (100)	20/20 (100)
5,000	32.56 (0.30)	32.20 (0.27)	20/20 (100)	20/20 (100)
50,000	28.70 (0.22)	28.23 (0.17)	20/20 (100)	20/20 (100)

Ct, cycle threshold; RT-qPCR, quantitative reverse transcription PCR; SARS-CoV-2, severe acute respiratory syndrome coronavirus 2.

### Comparison of performance of multiplex and single RT-qPCR

To confirm the sensitivity of the primer–probe sets (FAM, HEX, and Cy5 fluorophores) tested as single or multiplex reactions, as well as in comparison to the original single assay (FAM), we used nasopharyngeal swab and saliva samples from COVID-19 patients to detect SARS-CoV-2 RNA. The Ct values generated by the multiplex RT-qPCR were similar to those generated with FAM only or multicolor single RT-qPCR (Fig 1B; Table 2). These data indicated that our RT-qPCR with multicolor fluorophores under singleplex and multiplex conditions has similar performance for the detection of SARS-CoV-2 RNA as the currently utilized single RT-qPCR.

**Table 2:** Comparison of Ct values between single and multiplex RT-qPCR.

Sample	Single (only FAM)			Single			Multiplex		
	N1	N2	RP	N1	N2	RP	N1	N2	RP
NP_1	31.78	33.31	24.28	31.44	31.58	22.75	31.73	32.30	22.89
NP_2	20.73	22.19	27.29	20.25	19.75	25.69	20.34	19.96	25.20
Saliva_1	26.01	27.24	23.48	25.93	26.27	22.03	26.26	26.23	22.39
Saliva_2	30.58	32.04	21.69	31.15	31.33	20.59	31.41	32.08	20.94
P	31.68	33.4	ND	32.60	32.80	ND	32.17	32.17	ND
N	ND	ND	ND	ND	ND	ND	ND	ND	ND

Ct, cycle threshold; N, negative control; ND, not detected P, positive control (10^3^ virus copies/μl); RP, human RNase P; RT-qPCR, quantitative reverse transcription PCR.

### Comparison of single and multiplex assay sensitivity in clinical samples

To evaluate the accuracy of our RT-qPCR multiplex assay, we tested RNA extracted from nasopharyngeal swabs and saliva samples obtained from a total of 59 samples, including 38 SARS-CoV-2-positive inpatients and 21 SARS-CoV-2-negative healthcare workers. The results of our multiplex RT-qPCR were 100% sensitive as compared with single RT-qPCR (Fig 1C; Table 3). These data show that our multiplex RT-qPCR method could provide an alternative to the detection of SARS-CoV-2 by currently published single methods.

**Table 3:** The Ct values and results from the multiplex assay in clinical samples.

Sample	Sample type	Single (FAM)				Multiplex				Delta Ct (multiplex versus single)		
		N1	N2	RP	Result	N1	N2	RP	Result	N1	N2	RP
1	NP	32.29	32.96	23.99	Positive	33.01	33.06	23.62	Positive	0.72	0.10	−0.37
2	NP	29.65	30.78	25.51	Positive	30.50	30.58	25.00	Positive	0.85	−0.20	−0.51
3	NP	31.52	33.29	31.41	Positive	33.20	33.24	32.16	Positive	1.68	−0.05	0.75
4	NP	24.4	25.07	24.82	Positive	25.24	25.02	24.23	Positive	0.84	−0.05	−0.59
5	NP	19.57	20.24	23.94	Positive	19.58	19.13	22.20	Positive	0.01	−1.11	−1.74
6	NP	35.33	36.72	23.93	Positive	35.36	36.37	22.98	Positive	0.03	−0.35	−0.95
7	NP	34.46	36.03	26.27	Positive	35.37	35.34	25.46	Positive	0.91	−0.69	−0.81
8	NP	24.13	25.13	26.49	Positive	24.03	23.73	25.20	Positive	−0.10	−1.40	−1.29
9	NP	24.9	25.91	27.34	Positive	24.90	24.63	25.78	Positive	0.00	−1.28	−1.56
10	NP	31.31	32.41	28.67	Positive	31.21	31.12	27.64	Positive	−0.10	−1.29	−1.03
11	NP	26.07	27.02	26.82	Positive	25.83	25.58	25.09	Positive	−0.24	−1.44	−1.73
12	NP	34.15	36.31	27.34	Positive	35.15	35.45	26.65	Positive	1.00	−0.86	−0.69
13	NP	36.32	37.37	26.35	Positive	36.40	37.44	25.03	Positive	0.08	0.07	−1.32
14	NP	32.03	33.32	26.8	Positive	32.20	32.15	25.35	Positive	0.17	−1.17	−1.45
15	Saliva	34.39	35.99	24.38	Positive	34.35	34.91	22.80	Positive	−0.04	−1.08	−1.58
16	Saliva	32.34	33.77	24.19	Positive	32.09	32.47	22.41	Positive	−0.25	−1.30	−1.78
17	Saliva	30.98	32.23	21.33	Positive	33.02	34.14	21.02	Positive	2.04	1.91	−0.31
18	Saliva	15.63	16.36	22	Positive	15.73	15.13	21.36	Positive	0.10	−1.23	−0.64
19	Saliva	18.46	19.38	21.87	Positive	19.51	19.36	21.25	Positive	1.05	−0.02	−0.62
20	Saliva	26.86	27.86	21.82	Positive	27.53	27.42	21.29	Positive	0.67	−0.44	−0.53
21	Saliva	23.22	24.25	22.11	Positive	24.40	24.50	22.07	Positive	1.18	0.25	−0.04
22	Saliva	20.86	21.75	24.5	Positive	20.70	20.41	22.77	Positive	−0.16	−1.34	−1.73
23	Saliva	33.03	34.25	24.95	Positive	33.25	33.95	24.02	Positive	0.22	−0.30	−0.93
24	Saliva	20.77	21.7	25.25	Positive	20.53	20.23	23.29	Positive	−0.24	−1.47	−1.96
25	Saliva	33.17	34.56	21.53	Positive	35.29	37.28	20.58	Positive	2.12	2.72	−0.95
26	Saliva	33.46	35.2	28.07	Positive	33.82	34.23	27.22	Positive	0.36	−0.97	−0.85
27	Saliva	33.63	34.81	22.87	Positive	33.54	35.68	21.68	Positive	−0.09	0.87	−1.19
28	Saliva	20.59	22.09	22.83	Positive	25.92	25.16	21.54	Positive	5.33	3.07	−1.29
29	Saliva	25.24	26.79	20.72	Positive	25.89	26.31	19.26	Positive	0.65	−0.48	−1.46
30	Saliva	34.6	36.07	23.75	Positive	34.59	35.60	22.53	Positive	−0.01	−0.47	−1.22
31	Saliva	24.97	26.16	24	Positive	24.60	24.83	22.81	Positive	−0.37	−1.33	−1.19
32	Saliva	34.21	35.55	23.77	Positive	34.46	35.93	22.92	Positive	0.25	0.38	−0.85
33	Saliva	28.31	29.86	22.65	Positive	28.63	31.37	21.63	Positive	0.32	1.51	−1.02
34	Saliva	32.43	34.66	20.51	Positive	33.29	36.23	19.84	Positive	0.86	1.57	−0.67
35	Saliva	24.84	25.77	22.39	Positive	23.91	24.6	21.44	Positive	−0.93	−1.17	−0.95
36	Saliva	23.43	24.04	25.78	Positive	22.14	22.16	24.11	Positive	−1.29	−1.88	−1.67
37	Saliva	35.07	35.66	23.54	Positive	33.75	35.64	22.49	Positive	−1.32	−0.02	−1.05
38	Saliva	27.84	29.17	20.5	Positive	27.09	29.08	19.62	Positive	−0.75	−0.09	−0.88
39	NP	ND	ND	29.6	ND	ND	ND	28.78	ND			−0.82
40	NP	ND	ND	27.03	ND	ND	ND	26.09	ND			−0.94
41	NP	ND	ND	29.86	ND	ND	ND	29.24	ND			−0.62
42	NP	ND	ND	29.34	ND	ND	ND	28.50	ND			−0.84
43	NP	ND	ND	29.54	ND	39.02	ND	23.55	ND			−5.99
44	NP	ND	ND	27.9	ND	39.74	ND	26.83	ND			−1.07
45	NP	ND	ND	30.05	ND	39.87	ND	29.42	ND			−0.63
46	NP	ND	ND	27.86	ND	39.17	ND	26.87	ND			−0.99
47	NP	ND	ND	31.78	ND	39.03	ND	30.73	ND			−1.05
48	NP	ND	ND	28.68	ND	39.98	ND	27.59	ND			−1.09
49	NP	ND	ND	30.87	ND	38.99	ND	30.7	ND			−0.17
50	Saliva	ND	ND	24.56	ND	ND	ND	23.15	ND			−1.41
51	Saliva	ND	ND	25.35	ND	ND	ND	23.96	ND			−1.39
52	Saliva	ND	ND	24.9	ND	ND	ND	23.63	ND			−1.27
53	Saliva	ND	ND	26.38	ND	ND	ND	25.12	ND			−1.26
54	Saliva	ND	ND	22.93	ND	38.76	ND	22.63	ND			−0.30
55	Saliva	ND	ND	24.2	ND	38.72	ND	23.22	ND			−0.98
56	Saliva	ND	ND	23.27	ND	39.9	ND	22.16	ND			−1.11
57	Saliva	ND	ND	23.96	ND	39.11	ND	23.05	ND			−0.91
58	Saliva	ND	ND	22.38	ND	39.07	ND	24.2	ND			1.82
59	Saliva	ND	ND	27.58	ND	38.63	ND	26.46	ND			−1.12
P		31.78	34.00	ND		33.10	32.86	ND		1.32	−1.14	
**Average**										0.41	−0.29	−1.03
**SD**										1.13	1.17	0.89

Ct, cycle threshold; ND, not detected; NP, nasopharyngeal; P, positive control (10^3^ virus copies/μl); RP, human RNase P.

## Discussion

We improved an existing research single RT-qPCR method using the CDC primer–probe sets for multiplex RT-qPCR for molecular diagnostic testing of SARS-CoV-2. This multiplex RT-qPCR approach simultaneously detected the CDC-recommended 2 gene segments of SARS-CoV-2 RNA (N1 and N2) and the internal control human RNase P gene in a single reaction for research purposes. This method performed as well as the single RT-qPCR on clinical samples and was highly sensitive for detecting all target genes. Generally, an important consideration for this multiplex RT-qPCR approach is that cycling conditions may vary depending on qPCR machines, sample type, and target gene. We therefore recommend that when implementing new assays, primer and probe concentrations should be optimized to individual lab conditions.

The CDC primer and probe sets for SARS-CoV-2 testing are recommended for clinical testing in the US [2]. We reported sensitivity of CDC primer and probe sets compared with others from the Chinese Center for Disease Control and Prevention [5], Charité Institute of Virology, Universitätsmedizin Berlin [6], and Hong Kong University [7]. In single RT-qPCR, the CDC N2 primer set has a lower detection capability than the CDC N1 primers [3]. Our multiplex RT-qPCR assay also showed that N1 and N2 primer–probe sets had detection rates of 60% and 25%, respectively, at 50 virus copies per reaction (Table 1). While the analytical sensitivity is important to define for a given diagnostic test, very low viral copy numbers are unlikely to reflect infectious viral load [8].

The SARS-CoV-2 pandemic has already claimed the lives of over 400,000 people, and halted the global economy and changed our daily lives worldwide. Given the lack of available therapeutics or vaccines, we rely on public health measures such as testing, contact tracing, and quarantine. A rapid and accurate diagnostic test that is not cost prohibitive to identify infected individuals is urgently needed. Our multiplex RT-qPCR protocol described in this study provides rapid and highly sensitive detection of SARS-CoV-2 RNA for research purposes. In the future, Food and Drug Administration approval of such multiplex PCR techniques for clinical testing could provide a cost-effective solution to mass testing.

## Materials and methods

### Ethics statement

This study was approved by Yale Human Research Protection Program Institutional Review Boards (FWA00002571, Protocol ID. 2000027690). Informed consent was obtained from all enrolled patients and healthcare workers.

### Clinical samples

Clinical samples from SARS-CoV-2-positive inpatients (who were previously tested positive by a CLIA-certified laboratory prior to enrollment) and healthcare workers at Yale New Haven Hospital were collected as part of Yale’s IMPACT biorepository. RNA was extracted from nasopharyngeal and saliva samples using the MagMax Viral/Pathogen Nucleic Acid Isolation Kit (Thermo Fisher Scientific, Waltham, MA, US), according to a modified protocol [4].

### Control samples

Full-length SARS-CoV-2 RNA (WA1_USA strain from University of Texas Medical Branch; GenBank: MN985325) [9] was used as positive control for validation. Total RNA extracted from human embryonic kidney cell line 293T was used for detection of internal host gene control.

### Single and multiplex RT-qPCR

All reactions were performed on a CFX96 Touch instrument (Bio-Rad, Hercules, CA, US) using Luna Universal Probe One-Step RT-qPCR Kit (New England BioLabs, Ipswich, MA, US) according to the manufacturer’s protocol. A final reaction volume of 20 μl containing 5 μl of template was used. The following cycling conditions were applied: a cDNA synthesis step (10 min/55°C), a hold step (1 min/95°C), and subsequently 45 cycles of denaturation (10 s/95°C) and annealing/elongation (30 s/55°C). Nuclease-free water was used as the non-template control. The primer pairs and probes for single and multiplex RT-qPCR are shown in Table 4. We calculated the analytic efficiency of RT-qPCR assays tested with full-length SARS-CoV-2 RNA using the following formula:

$$E=100\times (10^{-\frac{1}{slope}}-1)$$

**Table 4:** Primers and probes for single and multiplex RT-qPCR.

Primer/probe name	Sequence (5′ → 3′)	Concentration (nM)		Ref.
		Single	Multiplex	
CDC N1 Forward	GACCCCAAAATCAGCGAAAT	500	400	[2]
CDC N1 Reverse	TCTGGTTACTGCCAGTTGAATCTG	500	400	
CDC N1 Probe	FAM-ACCCCGCATTACGTTTGGTGGACC-BHQ1	250	200	
CDC N2 Forward	TTACAAACATTGGCCGCAAA	500	400	[2]
CDC N2 Reverse	GCGCGACATTCCGAAGAA	500	400	
CDC N2 Probe	FAM-ACAATTTGCCCCCAGCGCTTCAG-BHQ1	250		
CDC N2 Probe	HEX-ACAATTTGC/ZEN/CCCCAGCGCTTCAG-IBFQ		200	
CDC RNase P Forward	AGATTTGGACCTGCGAGC	500	200	[2]
CDC RNase P Reverse	GAGCGGCTGTCTCCACAAGT	500	200	
CDC RNase P Probe	FAM-TTCTGACCTGAAGGCTCTGCGCG-BHQ1	250		
CDC RNase P Probe	Cy5-TTCTGACCTGAAGGCTCTGCGCG-IBRQ		100	

RT-qPCR, quantitative reverse transcription PCR.

### Analytical sensitivity

LOD was determined using full-length SARS-CoV-2 RNA. Viral RNA was 10-fold serially diluted in pooled nasopharyngeal swabs from SARS-CoV-2-undetected human samples at the following concentrations: 1, 10, 100, 1,000, and 10,000 copies/μl. The LOD was defined as the lowest RNA concentration detected in all of 20 replicates. Ct values over 40 were removed from analysis as non-detected.

### Data availability

All data associated with this manuscript are either included in the tables or made available upon request.

## Abbreviations

CDC: Centers for Disease Control and Prevention
COVID-19: coronavirus disease 2019
Ct: cycle threshold
LOD: limit of detection
RT-qPCR: quantitative reverse transcription PCR
SARS-CoV-2: severe acute respiratory syndrome coronavirus 2
